# Conformationally restricted short peptides inhibit human islet amyloid polypeptide (hIAPP) fibrillization†
†Electronic supplementary information (ESI) available: Experimental procedures, list of all the synthesized peptides and their % hIAPP fibrillization inhibition, MTT cytotoxicity assay, crystallization, details of X-ray structure determination, *in silico* docking of FGAΔFI with hIAPP, CD studies, Tables S1–S4, and Fig. S1–S6. CCDC 822015 and 904790. For ESI and crystallographic data in CIF or other electronic format see DOI: 10.1039/c3cc38982kClick here for additional data file.



**DOI:** 10.1039/c3cc38982k

**Published:** 2013-02-25

**Authors:** Aseem Mishra, Anurag Misra, T. Sri Vaishnavi, Chaitanya Thota, Madhvi Gupta, Suryanarayanarao Ramakumar, Virander Singh Chauhan

**Affiliations:** a International Centre for Genetic Engineering and Biotechnology , Aruna Asaf Ali Marg , New Delhi 110067 , India . Email: virander@icgeb.res.in ; Fax: +91-11-26742316 ; Tel: +91-11-26741358; b Department of Physics , Indian Institute of Science , Bangalore 560012 , India . Email: ramak@physics.iisc.ernet.in ; Tel: +91-80-22932312

## Abstract

hIAPP fibrillization implicated in Type 2 diabetes pathology involves formation of oligomers toxic to insulin producing pancreatic β-cells. We report design, synthesis, 3D structure and functional characterization of dehydrophenylalanine (ΔF) containing peptides which inhibit hIAPP fibrillization. The inhibitor protects β-cells from hIAPP induced toxicity.

## Abstract 2

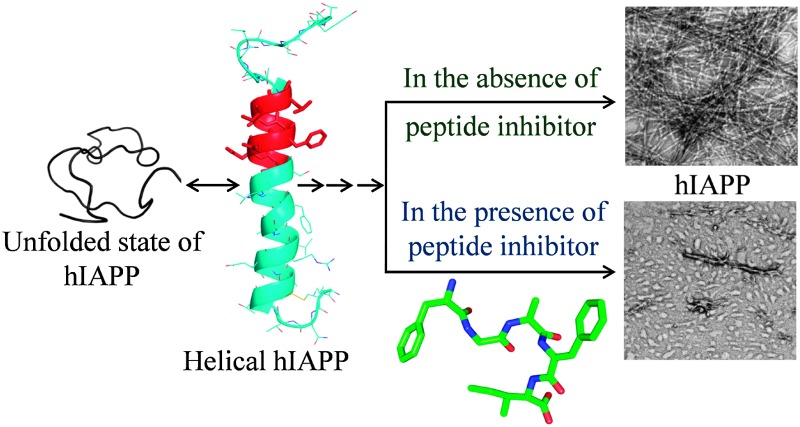
Inhibition of human islet amyloid polypeptide (hIAPP) fibrillisation by peptides incorporating a helicogenic amino acid, dehydrophenylalanine: implications for Type-2 diabetes.

Type 2 Diabetes Mellitus (T2DM) is one of the most prevalent endocrine disorders underlining the importance of developing molecular therapies to mitigate T2DM.^1^ It is characterized by a significant decrease in β-cell mass, insulin resistance and presence of amyloid plaques^2^ in which human islet amyloid polypeptide (hIAPP) is the major protein component.^3^ hIAPP is a 37-residue polypeptide co-secreted with insulin in β-cells of islets of Langerhans. A large amount of evidence favors its wide role in glucose metabolism.^4^ hIAPP is known to form amyloid fibrils with cross-beta structure,^5^ and amyloid deposits as the product of aggregation, but the process proceeds through oligomerization.^6,7^ It has been suggested that hIAPP oligomers of pore-like morphology are formed by association of helical monomers which then perform membrane fragmentation by pore formation.^8^ Thus, these prefibrillar oligomers are considered to be toxic and are implicated in β-cell dysfunction and death.^8b,9^ Hence, the impairment of oligomerization of helices by using designed small molecule inhibitors such as short peptides is a therapeutically relevant strategy for the prevention of T2DM. In this report, we show that two pentapeptides related to one of the core fibrillization regions of hIAPP inhibit fibril formation of hIAPP. Crystal structure analysis revealed an anion receptor ‘nest’ motif in these inhibitors, which based on computational studies was shown to interact with helical monomers of hIAPP. We also propose a model for fibrillization inhibition by these peptides.

Among the core fibrillization motifs/fragments of hIAPP,^10^ hIAPP_(22–27)_, *i.e.* NFGAIL, has been shown to form amyloid fibrils similar to those formed by the full-length polypeptide.^11^ Based on the motif hIAPP_(22–27)_, we designed several peptides as possible inhibitors of hIAPP fibrillization by strategically incorporating a non-natural amino acid α,β-dehydrophenylalanine (ΔF). ΔF is an analogue of phenylalanine with a double bond between C^α^ and C^β^ atoms and its presence induces β-turn in short peptides and helical secondary structures in longer peptides.^12^ Also, peptides containing ΔF resist enzymatic proteolysis,^13^ an added advantage for inhibitor design.

NFGAIL contains two β-favoring residues, F^23^ and I^26^, and their replacement with the helicogenic residue ΔF, individually or together, was a preferred choice for inhibitor design. I^26^ is an important residue; I^26^ → P mutation in full length hIAPP resulted in a hIAPP fibrillization inhibitor.^14^ Designed peptides (Table S1, ESI†) were synthesized using solid phase methods, purified on reverse phase HPLC and their identity confirmed by mass spectroscopy (ESI†). Fibrillization was quantified by the enhancement of thioflavin T (ThT) fluorescence upon its binding to fibrils. The % fibrillization inhibition activities are presented in Table S1 (ESI†).

I^26^ → ΔF mutation in the fibrillizing motif resulted in penta- and hexapeptides, FGAΔFL and NFGAΔFL, respectively. Neither of the two peptides showed β-sheet conformation and fibrillization property. ThT assay revealed (Table S1, ESI†) that FGAΔFL inhibited hIAPP fibrillization much more efficiently (75 ± 8%) than NFGAΔFL (7 ± 5%). Therefore, we focussed further studies on FGAΔFL. The fibrillization kinetics of hIAPP in the presence of the pentapeptide was studied. The exponential increase in ThT intensity, considered as a hallmark of fibril formation, was suppressed greatly when hIAPP was incubated with FGAΔFL in 1 : 5 molar ratio (Fig. 1a) suggesting that the peptide probably curtailed fibrillization at the stage of pre-fibrillar intermediates. Transmission electron microscopy (TEM) studies also confirmed that FGAΔFL significantly decreased hIAPP fibril formation (Fig. 1b and c).

**Fig. 1:**
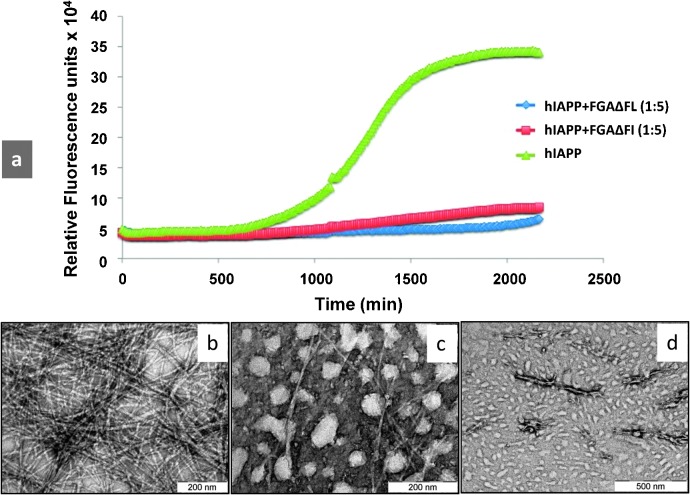
(a) Kinetics of hIAPP fibrillization in the presence and absence of inhibitors. Time course of amyloid formation monitored by fluorescence detected thioflavin-T binding: wild-type hIAPP alone (green) and coincubated with 5 M excess of the designed inhibitors FGAΔFL (blue) and FGAΔFI (red). Transmission electron micrographs of (b) hIAPP aged for 40 h, (c) incubated with FGAΔFL, and (d) FGAΔFI.

To explore the structure–function relationship, we determined the 3D structure of F^1^–G^2^–A^3^–ΔF^4^–L^5^ through X-ray crystallography (Table S2, ESI†). In the molecule G^2^ and A^3^ showed *α*_L_ and *α*_R_ conformations, respectively, and ΔF^4^ showed nearly helical conformation. Interestingly, a special structural motif ‘nest’ was observed in the molecule (Fig. 2). In this, two consecutive N-terminal residues (G^2^–A^3^) were positioned such that their main chain NH groups including free NH_3_^+^ formed a concave depression serving as an anion receptor, which interacted with terminal carboxyl oxygen from a symmetry related molecule. The ‘nest’ serves as the binding site of an ‘egg’ which is an atom or a group of atoms with full or partial negative charge.^15^ The peptide also showed a type-I β-turn formed by intramolecular N–H···O hydrogen bonding between L^5^_(*i*+3)_ → G^2^_(*i*)_ (Tables S3 and S4, ESI†).

**Fig. 2:**
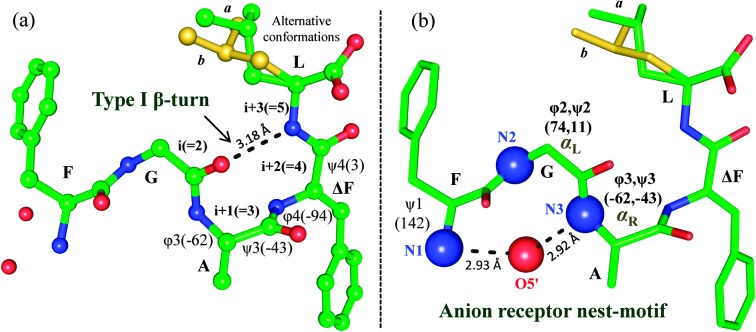
Molecular conformation of FGAΔFL. (a) Type-I β-turn and (b) anion receptor nest-motif. Leu side chain observed in two alternative conformations (*a*: green, *b*: yellow). (Details are given in ESI†.)

To investigate the possible modes of interaction of FGAΔFL with hIAPP, we performed molecular docking of FGAΔFL with the 3D structure of hIAPP (PDB: 2KB8)^16^ using AutoDock4.^17^ The best docked pose resulted in the binding energy of –6.47 kcal mol^–1^ (Fig. 3a). In the docked complex, FGAΔFL bound to the stretch which includes one of the core fibrillization motifs at the C-terminal half helical region *i.e.* SNNFGAIL (hIAPP_20–27_) with a shape complementarity value (Sc)^18^ of 0.83 indicating that hIAPP and the inhibitor have complementary binding surfaces. A helical wheel plot of hIAPP_13–30_ (Fig. 3b) shows that the face containing small sized residues (G and S) could easily be approached by the inhibitor. Docking studies suggested that the nest-motif formed by the FGA stretch of the pentapeptide interacted with the main chain and/or side chain carbonyl/hydroxyl oxygens from hIAPP to satisfy the hydrogen bond accepting potential of the motif. ΔF^4^ in FGAΔFL was involved in aromatic π–π stacking interaction with the hIAPP–F^23^ ring and two clusters of hydrophobic interactions were formed, F^1^L^5^ (peptide) & L^16^V^17^ (hIAPP) and ΔF^4^ (peptide) & F^23^L^27^ (hIAPP).

**Fig. 3:**
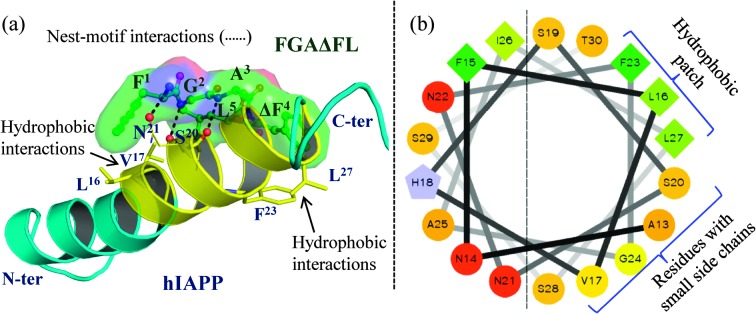
(a) The best docked pose of hIAPP with FGAΔFL. (b) Helical wheel diagram of hIAPP_13–30_ showing the hydrophobic patch and residues with small side chains which interact with FGAΔFL.

Circular dichroism (CD) spectra of hIAPP in the presence and absence of inhibitors at two different stages of the hIAPP fibrillization process *i.e.* when the hIAPP was in prefibrillar form (3 h old hIAPP) (Fig. S5, ESI†) and when it reached its fibrillar stage (96 h old hIAPP) (Fig. S6, ESI†) were recorded and plotted as additive and complex spectra. CD spectra remained unchanged when FGAΔFL was added to hIAPP when it had already attained β-form. In contrast, the addition of inhibitors at the prefibrillar state of hIAPP demonstrated noticeable differences in the spectra that may be because of inhibitor’s binding at the prefibrillar stage.

hIAPP is known to be highly cytotoxic to pancreatic cells.^4^ In order to test whether the FGAΔFL would reduce the cytotoxity of hIAPP we carried out cell viability assay. Results of the MTT (3-[4,5-dimethylthiazol-2-yl]-2,5-diphenyltetrazolium bromide) assay revealed that cytotoxic effects of hIAPP on cultured pancreatic rat insulinoma cells (RIN 5fm) showed a noticeable reduction in the presence of FGAΔFL. The inhibitor on its own did not show any cytotoxic effects on the cell line.

To further probe structural requirements involved in binding of the inhibitor with hIAPP, we also synthesized FGAΔFI, an analogue of the pentapeptide in which L^26^ was replaced with a β branched amino acid, I, and determined its 3D structure through X-ray crystallography (Fig. S1 and Table S2, ESI†). ThT binding assay showed that FGAΔFI was as effective as FGAΔFL in fibrillization inhibition (∼70% inhibition) (Table S1, ESI†). TEM of hIAPP incubated with FGAΔFI showed numerous vesicular structures without any significant amyloid fibers (Fig. 1d). It was gratifying to note that although FGAΔFI crystallized in a different space group *P*2_1_ from that of FGAΔFL (*i.e. P*2_1_2_1_2_1_) (Table S2, ESI†), it also had an anion receptor nest-motif and superposed with a backbone RMSD of 0.123 Å with FGAΔFL (Fig. S2, ESI†). Our *in silico* studies showed that both the peptides were able to dock to the monomeric helical state of hIAPP (ESI†). Perhaps, the interactions involving the anion receptor together with hydrophobic interactions play a role in the inhibitory activity of the pentapeptides. However, additional modes of interactions may also occur since hIAPP is a flexible molecule.

Based on our *in silico* studies, we propose a possible model to explain the inhibition of hIAPP fibrillization by the peptides (Fig. 4). The inhibitors seem to act by binding to the monomeric helical state of hIAPP at the hIAPP_20–27_ region which is one of the core fibrillization regions of hIAPP implicated in the nucleation dependent mechanism for oligomerization and initiation of beta-sheet formation during the fibrillization event.^19^ Binding of the inhibitor would stabilize the monomeric helical form of hIAPP and would decrease contact between helices coming in the way of helix–helix association. This would reduce the possibility of oligomer formation and fibrillization.

**Fig. 4:**
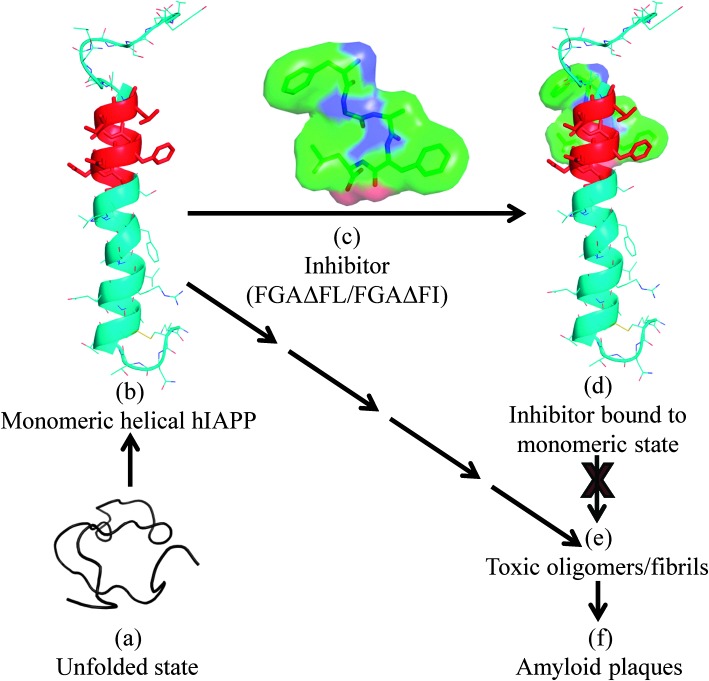
A proposed model for the inhibition of hIAPP fibrillization by FGAΔFL/FGAΔFI. (a) Unfolded state of hIAPP; (b) monomeric helical form of hIAPP (2KB8); (c) 3D structure of the inhibitor FGAΔFL, stick and surface representations; (d) inhibitor computationally docked with hIAPP; complex structure. The binding of inhibitors to hIAPP is expected to discourage the formation of toxic oligomers/fibrils (e), which in the final stage of aggregation result in amyloid plaques (f).

hIAPP fibrillization inhibitors include organic molecules,^20^ fragments of hIAPP^21,22^ as well as variants of native protein^14,23–25^ and its fragments.^26–28^ An approach in these studies was to disrupt amyloid formation by hIAPP and include peptides containing beta-breaker residues like Aib, Pro and N-methylated residues.^14,23,28^ In many cases, attention has been focused on targeting fibril formation by reducing β-sheet extension and assembly. Targeting amyloid fibrils may not be a useful strategy due to an adverse effect of rapidly increasing oligomers.^29^ Since FGAΔFL binds to hIAPP in the monomeric state, it could, in principle, shift the equilibrium away from the formation of oligomers/intermediates toxic to beta cells. Therefore, targeting the transient monomeric helical state and discouraging helix–helix association leading to the formation of oligomeric nuclei in the early events of self assembly, as outlined here, may be an attractive strategy for inhibitor design.

To conclude, though it was not anticipated, crystal structure analysis revealed that FGAΔFL and its analogue FGAΔFI harbour the anion receptor ‘nest’ motif. Both peptides dock with the helical form of hIAPP which may contribute to the inhibitory function of the peptides through their interaction with hIAPP in the core fibrillization region. These peptides effectively inhibit hIAPP fibrillization *in vitro* and it seems that these are unique examples of nest-motif containing peptides that inhibit fibrillization. In general, the approach described here may be applicable to targeting helices or helical intermediates and could be utilized in developing inhibitors useful, apart from T2DM, in other amyloid diseases including Alzheimer’s disease and Parkinson’s disease.^30^

We thank Dr Patole for cell culture work at NCCS, Pune, India, and CCD diffractometer facility at IISc. Aseem thanks Wellcome Trust–DBT India Alliance and Anurag thanks the University Grants Commission, India, for Research Fellowships. MG thanks Department of Science and Technology (DST), Govt. of India and ICGEB, VSC thanks Department of Biotechnology, Govt. of India and SR thanks DST for support. We thank the reviewers for their valuable comments.

## References

[cit1] Hossain P., Kawar B., El Nahas M. (2007). N. Engl. J. Med..

[cit2] Butler A. E., Janson J., Bonner-Weir S. (2003). Diabetes.

[cit3] Westermark P., Wernstedt C., Wilander E. (1987). Proc. Natl. Acad. Sci. U. S. A..

[cit4] Westermark P., Andersson A., Westermark G. T. (2011). Physiol. Rev..

[cit5] Wiltzius J. J., Sievers S. A., Eisenberg D. (2008). Protein Sci..

[cit6] Magzoub M., Miranker A. D. (2012). FASEB J..

[cit7] Laganowsky A., Liu C., Sawaya M. R. (2012). Science.

[cit8] Soong R., Brender J. R., Macdonald P. M. (2009). J. Am. Chem. Soc..

[cit9] Haataja L., Gurlo T., Huang C. J. (2008). Endocr. Rev..

[cit10] Mao X. B., Wang C. X., Wu X. K. (2011). Proc. Natl. Acad. Sci. U. S. A..

[cit11] Tenidis K., Waldner M., Bernhagen J. (2000). J. Mol. Biol..

[cit12] Rajashankar K. R., Ramakumar S., Chauhan V. S. (1992). J. Am. Chem. Soc..

[cit13] English M. L., Stammer C. H. (1978). Biochem. Biophys. Res. Commun..

[cit14] Abedini A., Meng F., Raleigh D. P. (2007). J. Am. Chem. Soc..

[cit15] Watson J. D., Milner-White E. J. (2002). J. Mol. Biol..

[cit16] Patil S. M., Xu S., Sheftic S. R. (2009). J. Biol. Chem..

[cit17] Morris G. M., Goodsell D. S., Halliday R. S. (1998). J. Comput. Chem..

[cit18] Lawrence M. C., Colman P. M. (1993). J. Mol. Biol..

[cit19] Wiltzius J. J. W., Sievers S. A., Sawaya M. R. (2009). Protein Sci..

[cit20] Hebda J. A., Saraogi I., Magzoub M. (2009). Chem. Biol..

[cit21] Scrocchi L. A., Chen Y., Waschuk S. (2002). J. Mol. Biol..

[cit22] Potter K. J., Scrocchi L. A., Warnock G. L. (2009). Biochim. Biophys. Acta.

[cit23] Yan L. M., Tatarek-Nossol M., Velkova A. (2006). Proc. Natl. Acad. Sci. U. S. A..

[cit24] Meng F., Raleigh D. P., Abedini A. (2010). J. Am. Chem. Soc..

[cit25] Cort J. R., Liu Z., Lee G. M. (2009). Protein. Eng., Des. Sel..

[cit26] Kapurniotu A., Schmauder A., Tenidis K. (2002). J. Mol. Biol..

[cit27] Tatarek-Nossol M., Yan L. M., Schmauder A. (2005). Chem. Biol..

[cit28] Gilead S., Gazit E. (2004). Angew. Chem., Int. Ed..

[cit29] Hebda J. A., Miranker A. D. (2009). Annu. Rev. Biophys..

[cit30] Sipe J. D., Amyloid Proteins: The β-Sheet Conformation and Disease, Wiley-VCH, Weinheim, 2005.

