# Supplementary material for: Conformationally restricted short peptides inhibit human islet amyloid polypeptide (hIAPP) fibrillization
Source: Chem Commun (Camb). 2013 Feb 25;49(26):2688–90. doi: 10.1039/c3cc38982k (PMC3684849; doi:10.1039/c3cc38982k)
Supplement: Supplementary file 1 [file CC-049-C3CC38982K-s001.pdf]

This journal is © The Royal Society of Chemistry 2013

Electronic Supplementary Information (ESI)

Conformationally restricted short peptides inhibit human islet amyloid polypeptide (hIAPP) Fibrillization

Aseem Mishra,‡[a] Anurag Misra,‡[b] T. Sri Vaishnavi,[a] Chaitanya Thota,[a] Madhvi Gupta,[a] Suryanarayanarao Ramakumar,*[b] and Virander Singh Chauhan*[a]

[a] International Centre for Genetic Engineering and Biotechnology, Aruna Asaf Ali Marg, New Delhi-110067, India
[b] Department of Physics, Indian Institute of Science, Bangalore-560012, India

‡ Both authors contributed equally, *Corresponding author

Table of Contents

[A] Experimental Section

Synthesis and Characterization of Peptides

Transmission Electron Microscopy (TEM)

Thioflavin T (ThT) binding fluorescence

MTT Cytotoxicity Assay on RIN-5fm

X-ray crystallography, Structure description and Molecular Docking

Circular Dichroism Spectroscopy

[B] Tables

Table S1. List of designed peptides and their percentage (%) hIAPP-fibrillization inhibition activity measured through ThT binding assay.

Table S2. Crystal data, data collection and structure refinement of FGAΔFL & FGAΔFI.

Table S3. Torsion angles (in degrees) for peptide FGAΔFL & FGAΔFI.

Table S4. The intermolecular and intramolecular hydrogen bonds observed in the crystal structures of FGAΔFL & FGAΔFI.

[C] Figures

Fig. S1. Molecular conformations of FGAΔFI with conserved Nest-motif and type-I β-turn.

Fig. S2. Superposed structures of FGAΔFL and FGAΔFI (with a backbone RMSD of 0.123 Å).

Fig. S3. Docked complex structure of hIAPP with FGAΔFI.

Fig. S4. TEM of hIAPP incubated with 10M excess of NΔFGAIL.

Fig. S5: CD spectra for the interaction between FGAΔFL and hIAPP during lag phase of hIAPP fibrillization.

Fig. S6: CD spectra for the interaction between FGAΔFL and hIAPP in fibrillar form i.e. in beta sheet form.

Main text Fig. 2 detailed caption: The figure depicts molecular conformation of FGAΔFL determined from the crystal structure. Figure 2(a) shows the molecule in ball and stick model where single molecule of FGAΔFL was observed in the asymmetric unit along with two water molecules. The C, N, O atoms in the molecule are represented by green, blue and red colour respectively. Leucine (L) side chain at the C-terminal was observed in two alternative conformations termed as *a* (green) & *b* (yellow). A dotted line shows H-bonding between i=5 to i=2 (i+3→i) which is a type I β-turn as shown in the figure 2a. Torsion angles for the residues involved in type I β-turn are mentioned. Figure 2(b) (nest-motif as anion receptor) is the stick representation of the peptide structure with three consecutive nitrogen atoms (N1, N2, N3) represented as spheres in blue forming nest and one spherical terminal oxygen atom (O5') an anion in red colour from a symmetry related molecule. Dotted lines show that O5' is H-bonded with N1 & N3 nitrogen

This journal is © The Royal Society of Chemistry 2013

atoms. Torsion angles for the residues forming nest (Gly in α_L form and Ala in α_R form) are mentioned.

[A] Experimental Section:

Synthesis and Characterization of Peptides: Peptides were synthesized using solid phase method using Fmoc (9-fluorenylmethyloxycarbonyl) chemistry, at a 0.5 mM scale. Wang's Resin was used to afford C-terminal carboxyl group. Couplings were performed by using diisopropylcarbodiimide. ΔF was introduced as a dipeptide block by dehydration of Fmoc-AA-DL-threo-β-phenyl serine (AA=Phe), using fused sodium acetate and freshly distilled acetic anhydride. Fmoc deprotection was performed with piperidine (20% in DMF). Peptide cleavage was achieved with 20 ml of 95:2.5:2.5 TFA: H_2O: TIS for 3 h, the resin filtered, filtrate concentrated, precipitated with cold ether, suspended in water, frozen and lyophilized to dryness. The crude peptides were purified by RP-HPLC. Peptide identities were confirmed using mass spectrometer. Lyophilized peptides were dissolved in DMSO/HFIP and diluted in phosphate buffer (pH 7.4) to investigate their effects on fibrillogenesis. To avoid any pre-aggregation, the stock solutions of the peptides were filtered through a 0.2 μ PTFE membrane filter. Fresh solutions were prepared for each experiment.

Transmission Electron Microscopy: Stock solutions (1 mM in HFIP) of $hIAPP_{(1-37)}$ were diluted to a final concentration of 40 μM either alone or in presence of 200 μM of synthetic peptides. The samples were allowed to fibrillize for 96 h at 37 °C without shaking. A 10 μl of the peptide solution was spotted on a 300 mesh carbon coated copper grid. The samples were stained with 1% UA and viewed on a FEI-Tecnai spirit T12 electron microscope operating at 120 kV.

Thioflavin T binding fluorescence: As described above, stock solutions (1 mM in HFIP) of hIAPP$_{(1-37)}$ were diluted to a final concentration of 40 µM either alone or in presence of 200 µM of designed peptides**.** Thioflavin T was added to a final concentration of 5 µM. The samples were allowed to fibrillize for 96 h at 37 °C without shaking (quiescent). Fluorescence intensities were measured at room temperature with excitation at 446 nm and emission at 480 nm using Perkin Elmer multi-label plate counter Victor 3. At least 8 replicates were taken for each sample. Percentage inhibition was calculated using the formula:

% Inhibition = [{Fluorescence Intensity (hIAPP) - Fluorescence Intensity (hIAPP mixed with synthetic peptide)}/ Fluorescence Intensity (hIAPP)] x 100

MTT Cytotoxicity Assay on RIN-5fm: The rat insulinoma cell line RIN-5fm was cultured in RPMI 1640. Cells were plated in 96-well plates at a density of 5 x 10^5 cells/ml (100 µl/well) and incubated for 20 h (37 °C, humidified atmosphere with 5% CO_2). hIAPP$_{(1-37)}$ was dissolved in DMSO (1 mM of stock solution) and then diluted in cell culture media to a final concentration of 1 to 10 µM. The sample was kept for aging (72 h) prior to addition to the cells. The cells were then incubated with hIAPP fibrils alone or treated with FGAΔFL for 20 h. Cell viability was estimated through MTT assay.

X-ray crystallography: Single crystals of peptides FGAΔFL (**1**) and FGAΔFI (**2**) ($C_{29}H_{37}N_5O_6$, M_w = 552; crystal size: 0.40 x 0.07 x 0.05 and 0.20 x 0.04 x 0.01 mm^3 respectively) were obtained by controlled evaporation under oil (mixture of silicon oil and paraffin oil in 1:1 ratio) in micro-batch method using crystallization conditions containing 0.02 M Calcium chloride dihydrate, 0.1 M Sodium acetate trihydrate (pH

4.6) and MPD 30 % (v/v), and 0.2 M Ammonium sulphate, 0.1M Sodium cacodylate trihydrate (pH 6.5), PEG 8000- 30 % (w/v) respectively. The peptide **1** crystallized in orthorhombic space group $P2_12_12_1$ whereas peptide **2** crystallized in monoclinic space group $P2_1$ (Table S2). Both the peptides crystallized as single zwitterionic molecule per asymmetric unit with two water molecules. X-ray intensity data sets were collected at 100 K temperature on a Bruker AXS KAPPA APEX II CCD diffractometer (Mo Kα radiation, λ=0.71073 Å, ω & Φ scan). Both the structures were solved by direct methods by using SHELXS-97 program[1] and were refined against F^2, with the full-matrix least-squares methods employed in SHELXL-97 program.[2] In both the data sets, solvent water molecules were located from difference Fourier maps. After the convergence of isotropic refinement, anisotropic refinement was carried out for all non-hydrogen atoms, except for the Leu^4 side chain atoms which were exhibiting positional disorder in crystals of **1**. The Leu^4 side chain atoms (C^{β}, C^{γ}, $C^{\delta1}$ and $C^{\delta2}$) of the peptide **1** were refined in two positions with final occupancy ratio stabilizing at 0.53:0.47. And also, both the aromatic rings of Phe^1 and ΔPhe^4 were showing disorder for their bond lengths and bond angles in peptide **1**. Therefore, distance constraints were used during the refinement to optimize the bond lengths and bond angles of all the disordered groups in **1**. The hydrogen atoms in both the peptides were fixed geometrically in the idealized positions and were refined as riding over the atoms to which they are covalently bonded. For the peptide **2,** water hydrogens were located through difference Fourier map and refined isotropically. Refinement for both the peptides converged at the agreement factors shown in Table S2.

Circular Dichroism Spectroscopy: All CD experiments were carried out at room temperature in a 10 mM Phosphate buffer (pH 7.0) and spectra (average of four scans)

were collected using a Jasco-810 spectropolarimeter and quartz cuvette with a path length of 1 mm. CD spectra were collected between 195 to 300 nm at 0.2 nm intervals with a response time of 8 sec. CD spectra (mean residue ellipticities [θ]) were presented after subtracting the spectra of buffer alone.

To a 3 h aged hIAPP preparation equimolar (4 µM) FGAΔFL was added. The far-UV region represented primary contributions from hIAPP whereas the near UV region represented the ΔF chromophore of the inhibitor peptide. The additive spectra in both the cases represent the sum contribution of hIAPP and the inhibitor without any physical interaction (using a tandem cuvette) while the complexed spectra indicates the combined spectra of hIAPP and the inhibitor after 30 min of physical interaction.

Description of the crystal structures of FGAΔFL & FGAΔFI and their docking with helical hIAPP:

The crystallographic details of the peptide FGAΔFL are presented in Table S2. In the peptide 3D structure G^2 and A^3 show α_L and α_R conformations with torsion angles φ, ψ as (74º, 11º), (-62º, -43º) respectively; ΔF^4 shows nearly helical conformation (-94º, 3º). Important torsion angles for the peptide are listed in Table S3. All the peptide units are in *trans* conformation and as expected, the ΔF side chain is planar as indicated by the angles χ^1 = -3˚ & χ^2 = -39˚ (Table S3). An interesting conformational motif occurred in the structure (Fig. 3; main text) with the two consecutive N-terminal residues (G^2-A^3) having assumed enantiomeric conformations (φ_2, ψ_2 = 74º, 11º), (φ_3, ψ_3 = -62º, -43º) such that their main chain NH groups including free NH_3^+ group form a concave depression serving as an anion receptor. This geometrical feature where the backbone of successive residues assumes enantiomeric conformations and binds anions, termed as 'nest', is often found at functionally important regions in proteins, well-known examples being the P loop phosphate binding motif that occurs in G

This journal is © The Royal Society of Chemistry 2013

proteins and kinases, the oxy-anion hole in serine proteases, and fusion peptide region of influenza haemagglutinin.[3] The 'nest' serves as the binding site of an 'egg' which is an atom or a group of atoms with full or partial negative charge.[3c] The peptide also showed a type-I β-turn (Table S3 & S4) formed by intramolecular N-H···O hydrogen bonding between L^5 (i+3) → G^2 (i) (Table S3 & S4). To study 3D molecular interactions between the FGAΔFL and hIAPP, we performed molecular docking using AutoDock4.[4] We considered the NMR derived structure (PDB: 2KB8)[5] to be representative of the alpha helical structure transiently sampled by hIAPP in the solution state, prior to amyloid formation. Using this helical structure of hIAPP, peptide-ligand (hIAPP) interactions were modelled with the Lamarckian genetic algorithm with the grid box spanning the entire peptide.[4a] The best ligand pose was selected from the clusters with the highest occurrence and the lowest energy (Fig. 4a; main text). Binding energies ranged from -6.37 to -6.47 kcal/mol. In the docked complex, pentapeptide was found to bind at the C-terminal half helical region i.e. at SNNFGAIL ($hIAPP_{20-27}$) which is known to be a core fibrillization motif. Similar results were obtained when we used a recent NMR structure of hIAPP for the docking.[6] The helical wheel plot of $hIAPP_{13-30}$ (Fig. 4b; main text) also seemed to indicate that the face containing small sized residues (Gly and Ser) could be approached by the inhibitor. In a complex of hIAPP and inhibitor, the shape complementarity value (Sc)[7] was calculated as 0.83 which also suggest strong surface compatibility for effective binding to the helical form of hIAPP. Fig. 4a (main text) showed the interactions observed between FGAΔFL-hIAPP complex. Molecular docking suggested that the Nest-motif formed by FGA stretch of the pentapeptide was amenable to interact with the main chain and/or side chain carbonyl/hydroxyl oxygen to satisfy hydrogen bond accepting potential of the nest-motif with three hydrogen

This journal is © The Royal Society of Chemistry 2013

bonds F → N^{21}, G → S^{20} and A → S^{20}; S^{20} appears to be an important residue for inhibitor binding. Interestingly, the situation in which S^{20} is replaced by an even smaller residue G i.e. S20G mutation in hIAPP is associated with an increased risk of development of T2DM.[8] Thus it may be seen that the inhibitor peptide FGAΔFL interacts with hIAPP in a region which includes the important residue S^{20}. The ΔPhe^4 in FGAΔFL is seen to be involved in aromatic π-π stacking interaction with IAPP-Phe^{23} ring. In hydrophobic interactions two clusters were formed, F^1L^5 (peptide) & $L^{16}V^{17}$ (IAPP) and ΔF^4 (peptide) & $F^{23}L^{27}$ (IAPP).

The peptide FGAΔFI, a different but sequence similar peptide of FGAΔFL was also crystallized and its 3D structure was solved by X-ray crystallography (Table S2). The new design crystallized in different space group ($P2_1$) while retaining the same structural motif i.e. nest-motif, as discussed above. Table S1, S2, S3, S4 show comparative data for the peptide FGAΔFI with FGAΔFL. Also, the backbone RMSD was found to be 0.123 Å when both the structures were superposed (Fig. S2) establishing structural similarity between FGAΔFL and FGAΔFI. In a complex of hIAPP and the inhibitor FGAΔFI (Fig. S3), the shape complementarity value (Sc)[7] was calculated as 0.82 which infer strong surface compatibility for effective binding to the helical form of hIAPP. Similar nest-motif interactions and hydrophobic interactions were observed, and the lowest binding energy for this peptide was calculated as -5.56 kcal/mol. This shows that comparatively FGAΔFL is a better inhibitor than FGAΔFI.

Softwares for figure preparation

Pymol[9] as well as Microsoft Office PowerPoint 2007 were used to generate publication quality figures in main text as well as in supplementary information.

This journal is © The Royal Society of Chemistry 2013

[B] Tables

Table S1. List of designed peptides and their percentage (%) hIAPP-fibrillization inhibition activity measured through ThT binding assay.

**Sequence**	**% Inhibition of hIAPP fibrillization**	
	**Mean**	**Std. Dev (±)**
NΔFGAIL	47.36	5.32
NFGAΔFL	7.69	5.21
**FGAΔFL**	**75.91**	**8.45**
**FGAΔFI**	**70.63**	**6.29**
FGAUL	-52.24	16.23
FGAPL	4.01	2.36
NΔFGAΔFL	11.54	5.32

Table S2. Crystal data, data collection and structure refinement of FGAΔFL and FGAΔFI

	**FGAΔFL**	**FGAΔFI**
Formula	$C_{29}H_{37}N_5O_6.2H_2O$	$C_{29}H_{37}N_5O_6.2H_2O$
Mr	551.6+36	551.6+36
Solvent of crystallization	0.02 M Calcium chloride dihydrate; 0.1M Sodium acetate trihydrate; pH 4.6; MPD-30 % (v/v)	0.2 M Ammonium sulphate; 0.1M Sodium cacodylate trihydrate; pH 6.5; PEG 8000- 30 % (w/v)
Crystal system	Orthorhombic	Monoclinic
Space group	$P2_12_12_1$	$P2_1$
a [Å]	8.9951(9)	12.8946 (23)
b [Å]	13.0144(12)	9.0534 (16)
c [Å]	27.7521(24)	13.0895 (23)
β [°]	90	104.071 (9)
V [Å^3]	3248.82 (5)	1482.22 (35)
Z	4	2
Dx[g cm^{-3}]	1.19	1.32
μ ($Mo_{K\alpha}$)[mm^{-1}]	0.088	0.097
λ [Å]	0.71073	0.71073
Crystal size[mm^3]	0.40 x 0.07 x 0.05	0.20 x 0.04 x 0.01
Crystal shape, colour	Rod, colourless	Rod, colourless
Temperature[K]	100	100
Scan type/width[°]	Φ and ω/0.5	Φ and ω/0.5
Frame exposure time[s]	30	60
Reflections collected	59104	11784
Independent reflections	4703	4024
Observed reflections	2581 [|Fo|>4σ(|Fo|)]	2612 [|Fo|>4σ(|Fo|)]
θ_{range}[°]	1.5 - 23.3	1.6 - 24.5
Resolution [Å]	1.05	0.9
R	0.06 [|Fo|>4σ(|Fo|)]	0.05 [|Fo|>4σ(|Fo|)]
wR2	0.183	0.108
Goodness of fit	1.032	0.978
Data/parameters	2581/377 = 6.85	2612/396 = 6.6
Structure solution/refinement	SHELXS97/SHELXL97(Full matrix least square refinement on |Fo|^2)	SHELXS97/SHELXL97(Full matrix least square refinement on |Fo|^2)
$\Delta\rho_{max}/\Delta\rho_{min}$[e Å^{-3}]	0.381/-0.282	0.210/-0.235
**CCDC number**	**822015**	**904790**

This journal is © The Royal Society of Chemistry 2013

Table S3. Torsion angles (in degrees) for peptides FGAΔFL and FGAΔFI.

	**FGAΔFL**					**FGAΔFI**				
Angles	*Phe*	*Gly*	*Ala*	*ΔPhe*	*Leu**	*Phe*	*Gly*	*Ala*	*ΔPhe*	*Ile*
φ_i		74	-62	-94	-63		80	-61	-89	-62
ψ_i	142	11	-43	3		142	7	-38	-2	
ω_i	175	-170	179	-179		171	-171	-178	-178	
$\chi_i^{1,1}$	-168			-3	-174/-81	174			-5	-66
$\chi_i^{1,2}$										170
$\chi_i^{2,1}$	97			-39	71/170	41			-31	174
$\chi_i^{2,2}$	-82			143	-168/-66	-141			152	

*Leu side chain in two alternative conformations in FGAΔFL

Table S4. The intermolecular and intra-molecular hydrogen bonds observed in the crystal structures of FGAΔFL and FGAΔFI. As may be seen the nest region acts as an anion receptor in both the peptides

	Donor (D)	Acceptor (A)	D···A(Å)	H···A(Å)	D-H···A(°)	Symmetry code
**FGAΔFL**	N1	O2W	2.778	1.90	171	x,y,z
	**N5**	**O2'**	**3.180(a)**	**2.38**	**154**	**x,y,z**
	N1	O5"	2.869	2.01	161	x,+y+1,+z
	**N1**	**O5'**	**2.926(b)**	**2.21**	**138**	**x+1/2,-y+1/2,-z**
	**N3**	**O5'**	**2.915(b)**	**2.11**	**157**	**x+1/2,-y+1/2,-z**
	N2	O3'	2.933	2.34	126	x+1/2,-y+1/2,-z
	N4	O2'	2.938	2.16	151	x+1/2,-y+1/2,-z
	N1	O1W	3.287	2.57	138	x-1/2,-y+1/2+1,-z
	C4D1	O3'	3.342	2.51	150	x,y,z
	C5B'	O2'	3.690	2.61	163	x,y,z
	C2A	O3'	3.024	2.35	126	x+1/2,-y+1/2,-z
	Donor (D)	Acceptor (A)	D···A(Å)	H···A(Å)	D-H···A(°)	Symmetry code
**FGAΔFI**	N1	O1W	2.765	1.88	171	x,y,z
	**N5**	**O2'**	**3.095(a)**	**2.28**	**158**	**x,y,z**
	N1	O5"	2.889	2.01	171	x,+y,+z+1
	**N1**	**O5'**	**2.969(b)**	**2.27**	**135**	**-x+1,+y+1/2,-z**
	**N3**	**O5'**	**2.916(b)**	**2.08**	**163**	**-x+1,+y+1/2,-z**
	N2	O3'	2.878	2.28	127	-x+1,+y+1/2,-z
	N4	O2'	2.919	2.16	147	-x+1,+y+1/2,-z
	N1	O2W	3.255	2.52	140	-x+1,+y+1/2,-z+1
	C4D1	O3'	3.434	2.61	148	x,y,z
	C3B	O1w	3.544	2.72	144	-x+1,+y-1/2,-z+1
	C2A	O3'	3.004	2.36	123	-x+1,+y+1/2,-z
	C1A	O4'	3.757	2.79	168	-x+1,+y+1/2,-z
	O2W	O5"	2.788	1.91	171	x,+y,+z+1
	O1W	O1'	2.906	2.23	172	-x+1,+y+1/2,-z+1
	O1W	O2W	2.805	1.89	169	x,+y+1,+z
	O2W	O4'	2.753	1.91	177	-x+1,+y-1/2,-z

(a) Type-I β-turn (b) hydrogen bonds for Nest-egg interactions

[C] Figures

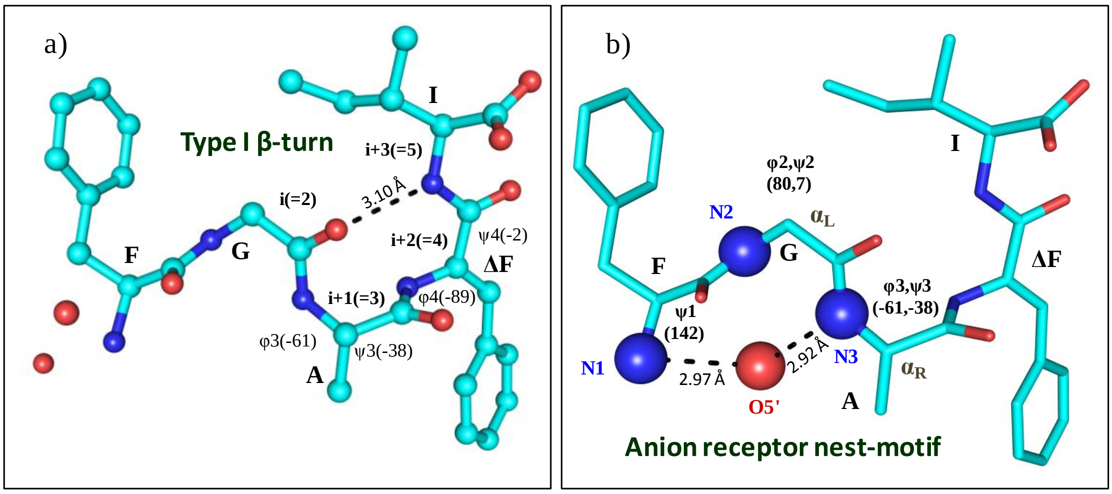

Fig. S1. Molecular conformations of FGAΔFI with conserved nest-motif and type I β-turn.

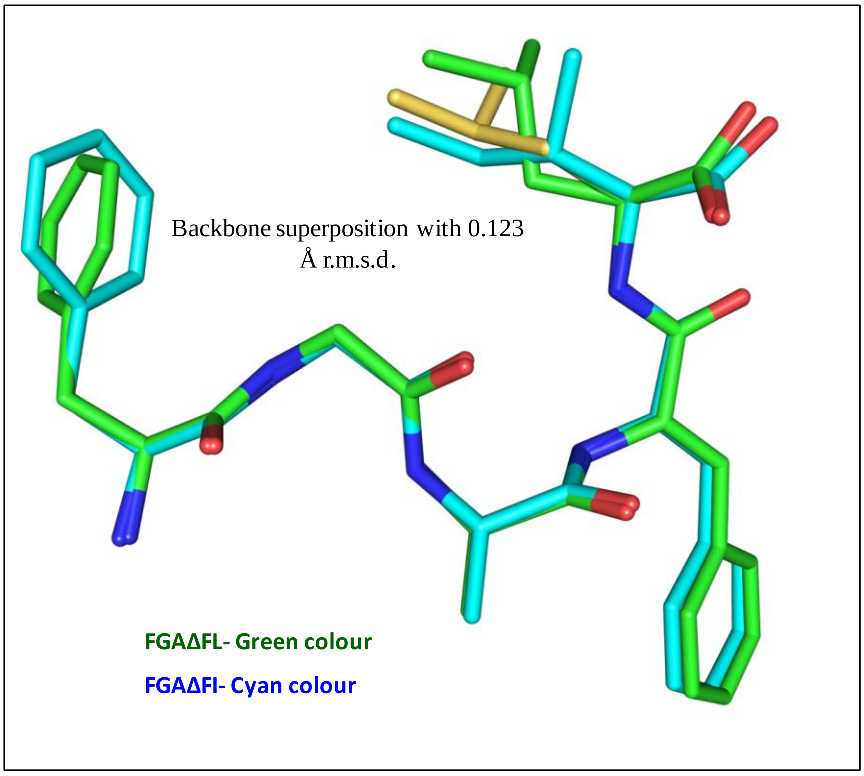

Fig. S2. Superposed structures of FGAΔFL and FGAΔFI (with a backbone r.m.s.d. of 0.123 Å).

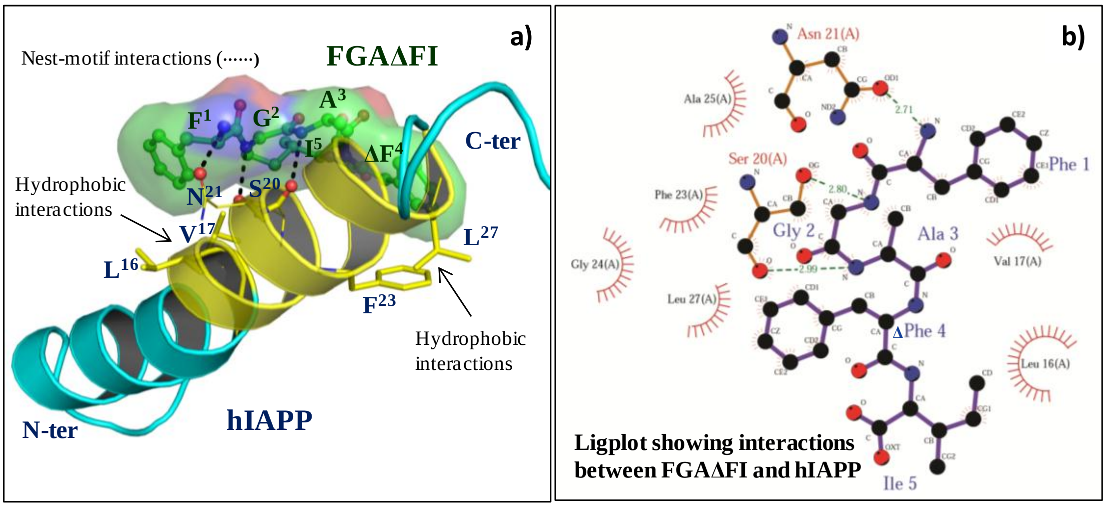

Fig. S3. Docked complex structure of hIAPP with FGAΔFI. It may be noticed that peptide Nitrogens make hydrogen bonds with some of the backbone oxygens (a). Ligplot of the docked complex is also shown (b).

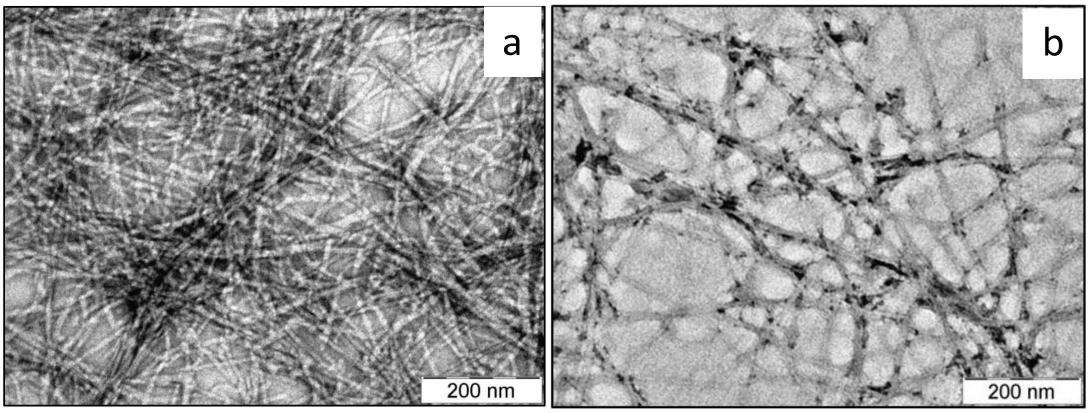

Fig. S4. TEM of a) hIAPP alone and b) hIAPP incubated with 10 M excess of NΔFGAIL for 96 h. This peptide showed reasonable inhibition in Thioflavin T assay. However, the occurrence of fibrils in the preparation suggest that NΔFGAIL does not inhibit fibrillization of hIAPP, rather leads to formation of a fibrillar form that has low affinity of Thioflavin T staining.

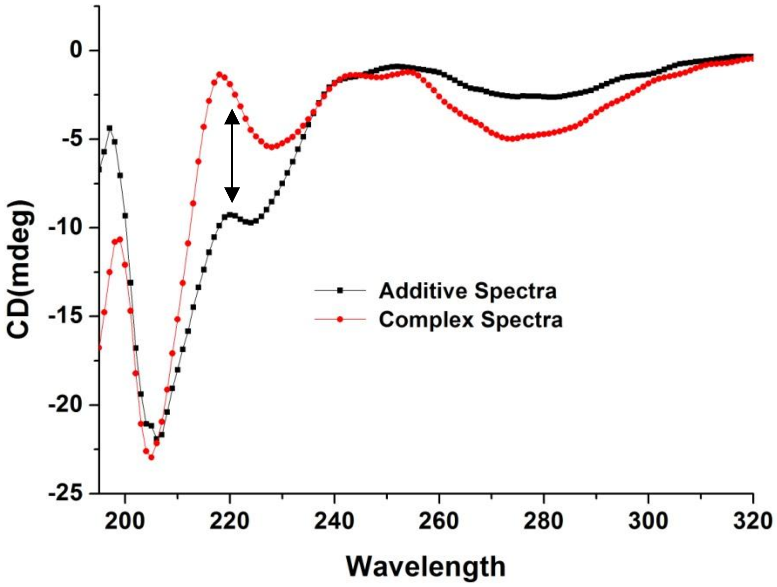

Fig. S5. The CD spectra of hIAPP in helical form interacting with FGAΔFL (additive Spectra indicates the sum signature of hIAPP and FGAΔFL physically separated from each other; Complex spectra indicates the sum signature after the components kept for 30 m of physical interaction.) Arrow indicate region of spectral differences.

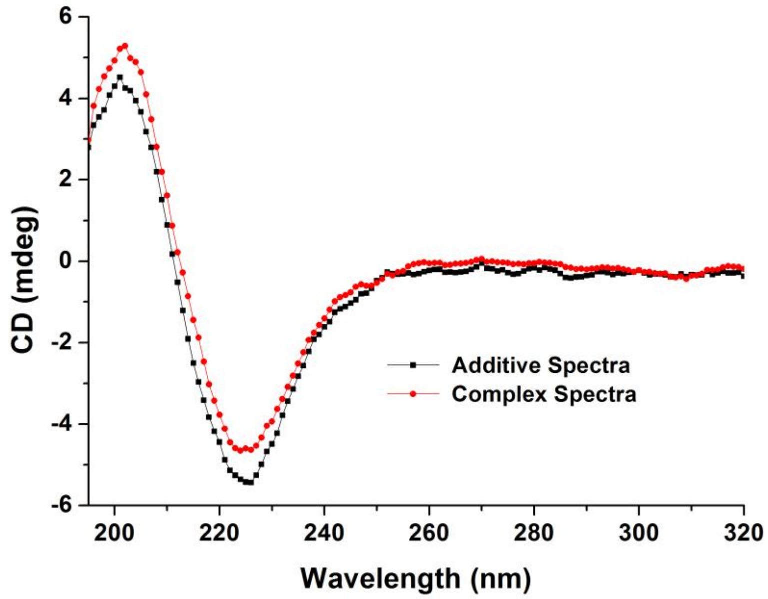

Fig. S6. Interaction between FGAΔFL and hIAPP in beta sheet form. The CD spectra of hIAPP (4 µM; 96 h) in beta sheet form interacting with FGAΔFL (additive Spectra indicates the sum signature of hIAPP and FGAΔFL physically separated from each other in a tandem cuvette; Complex spectra indicates the sum signature after 30 min of physical interaction of both the components.)

Supplementary References:

[1] G. M. Sheldrick, SHELXS-97, *A program for automatic solution of crystal structures, University of Gottingen, Gottingen*, 1997.

[2] G. M. Sheldrick, SHELXL-97, *A program for crystal structure refinement, University of Gottingen, Gottingen*, 1997.

[3] (*a*) J. D. Watson and E. J. Milner-White, *J. Mol. Biol.*, 2002, **315**, 171. (*b*) J. D. Watson and E. J. Milner-White, *J. Mol. Biol.*, 2002, **315**, 183. (*c*) D. Pal, J. Suhnel and M. S. Weiss, *Angew. Chem. Int. Ed.*, 2002, **41**, 4663. (*d*) E. J. Milner-White, J. W. Nissink and F. H. Allen, *et al*., J. *Acta. Crystallogr. D Biol. Crystallogr.*, 2004, **60**, 1935. (*e*) Rudresh, S. Ramakumar and U. A. Ramagopal, *et al*., *Structure*, 2004, **12**, 389.

[4] (*a*) G. M. Morris, D. S. Goodsell, R. S. Halliday, R. Huey, W. E. Hart, R. K. Belew and A. J. Olson, *J. Comput. Chem.*, 1998, **19**, 1639-1662. (*b*) G. M. Morris, R. Huey, W. Lindstrom, M. F. Sanner, R. K. Belew, D. S. Goodsell and A. J. Olson, *J. Comput. Chem.*, 2009, **30**, 2785-2791.

[5] S. M. Patil, S. Xu, S. R. Sheftic and A. T. Alexandrescu, *J. Biol. Chem.*, 2009, **284**, 11982-11991.

[6] R. P. Nanga, J. R. Brender, S. Vivekanandan and A. Ramamoorthy, *Biochim Biophys Acta*, 2011, **1808**, 2337-2342.

[7] M. C. Lawrence and P. M. Colman, *J. Mol. Biol.*, 1993, **234**, 946-950.

[8] S. Seino, *Diabetologia*, 2001, **44**, 906-909.

[9] *The PyMOL Molecular Graphics System, Version 1.5.0.4 Schrödinger, LLC.*
